# Role of the Cysteinyl Leukotrienes in the Pathogenesis and Progression of Cardiovascular Diseases

**DOI:** 10.1155/2017/2432958

**Published:** 2017-08-28

**Authors:** Francesca Colazzo, Paolo Gelosa, Elena Tremoli, Luigi Sironi, Laura Castiglioni

**Affiliations:** ^1^Centro Cardiologico Monzino IRCCS, Via Carlo Parea 4, 20138 Milan, Italy; ^2^Department of Pharmacological and Biomolecular Sciences, University of Milan, Via Giuseppe Balzaretti 9, 20133 Milan, Italy

## Abstract

Cysteinyl leukotrienes (CysLTs) are potent lipid inflammatory mediators synthesized from arachidonic acid, through the 5-lipoxygenase (5-LO) pathway. Owing to their properties, CysLTs play a crucial role in the pathogenesis of inflammation; therefore, CysLT modifiers as synthesis inhibitors or receptor antagonists, central in asthma management, may become a potential target for the treatment of other inflammatory diseases such as the cardiovascular disorders. 5-LO pathway activation and increased expression of its mediators and receptors are found in cardiovascular diseases. Moreover, the cardioprotective effects observed by using CysLT modifiers are promising and contribute to elucidate the link between CysLTs and cardiovascular disease. The aim of this review is to summarize the state of present research about the role of the CysLTs in the pathogenesis and progression of atherosclerosis and myocardial infarction.

## 1. Introduction

The leukotrienes (LTs) are lipid mediators belonging to a large family of molecules named eicosanoids—from the Greek word “eicosa” meaning 20—as they are generated from the arachidonic acid (AA), a carbon-20 polyunsaturated fatty acid, through the 5-lipoxygenase (5-LO) pathway [1, 2].

The synthesis of LTs begins with the cleavage of AA from the glycerol-phospholipids present into the cellular nuclear membrane. The 5-LO, with the aid of the accessory 5-LO-activating protein (FLAP), catalyzes the conversion of AA to 5-hydroperoxyeicosatetraenoic acid (5-HETE) and then to leukotriene A4 (LTA4) [3, 4], an unstable intermediate, which can be either metabolized by LTA4 hydrolase to LTB4, a potent chemoattractant, or conjugated to glutathione by LTC4 synthase (LTC4S) producing the cysteinyl LTs (CysLTs: LTC4, LTD4, and LTE4) [5].

The LTs exert their actions through interaction with specific 7-transmembrane G-protein-coupled cell surface receptors, BLT1 and BLT2, representing the high and low-affinity receptor for LTB4, respectively, and CysLT_1_ receptor (CysLT_1_R) and CysLT_2_ receptor (CysLT_2_R) activated by the CysLTs [6, 7] plus a recently discovered LTE4-specific receptor known as CysLT_E_R that was identified in CysLT_1_R/CysLT_2_R double-deficient mice [8]. The CysLTs present a different order of affinity for CysLT_1_R and CysLT_2_R. In detail, the rank of affinity toward CysLT_1_R is LTD4 > LTC4 > LTE4 whereas for CysT_2_R is LTC4 = LTD4 >> LTE4 [9, 10]. GPR17 and GPR99, recently identified, may also be additional receptors for LTD4/LTC4 [11] and LTE4, respectively [12]; moreover, LTE4 has been reported to upregulate COX-2 through the PPAR*γ* receptor in mast cells [13], as well as to bind the P2Y12 receptors [14]. As better detailed below, the CysLTs are synthetized by different cells and released in their extracellular space in response to several stimuli.

**Figure 1:**
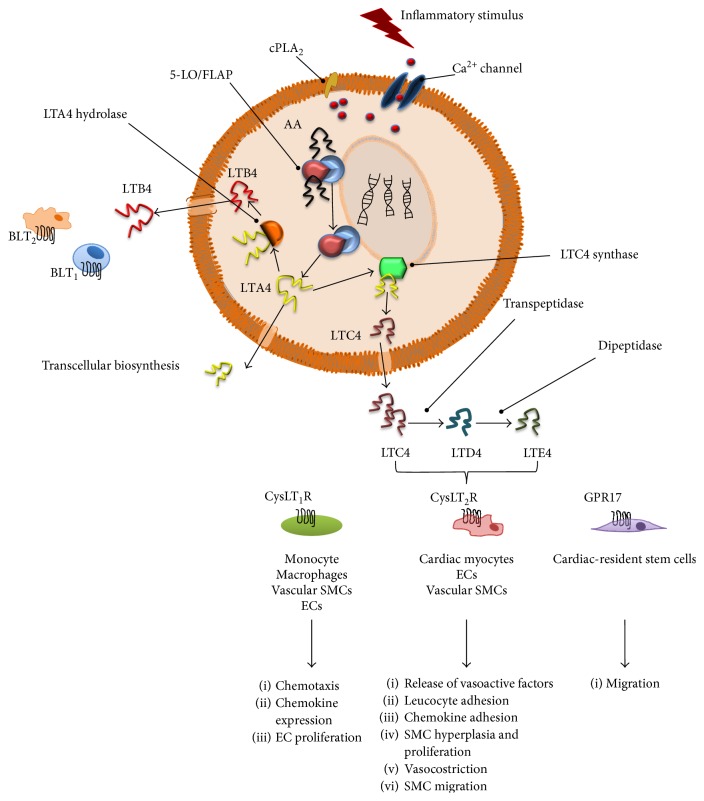
The 5-LO pathway: biosynthesis, signaling, and effect on cardiovascular system. 5-Lipoxygenase (5-LO), leukotriene (LT), cytosolic phospholipase A2 (cPLA_2_), arachidonic acid (AA), 5-LO-activating protein (FLAP), multidrug resistance protein-1 (MRP1), endothelial cells (ECs), and smooth muscle cells (SMCs).

The effects of CysLTs in the cardiovascular system are established and suggest the existence of a solid link between the 5-LO pathway and cardiovascular diseases (CVDs) (Figure 1).

This review will focus on current knowledge about the involvement of the CysLTs in atherosclerosis and myocardial infarction and on the effects mediated by the CysLT modifiers on the disease progression.

## 2. CysLT Actors in Cardiovascular System

Atherosclerosis and myocardial infarction are vascular pathologies characterized by inflammation. The eosinophils, basophils, mast cells, and macrophages, major effector cells of innate immunity, possess the integral membrane protein LTC4S [15] and are competent in synthesizing CysLTs in response to biological and nonbiological stimuli [16, 17]. Intriguing, cells unable to produce LTA4, such as vascular endothelial cells [18], platelets [19], but also mast cells [20], blood peripheral monocytes [21], human airway epithelial cells [22], alveolar macrophages [23], kidney-derived endothelial cells [24], keratinocytes [25], and chondrocytes [26], can use LTA4 generated from the surrounding cells (such as neutrophils) to produce LTC4 and the other CysLTs but also LTB4. This process, called transcellular biosynthesis, could generate high concentrations of CysLTs at the local level, affecting organ function [27].

The CysLT_1_R and CysLT_2_R present distinct tissue and cellular pattern expression only partially overlapping [28]. Regarding the cardiovascular system, the expression of the CysLT_1_R is hardly detectable [9, 29, 30], while that of the CysLT_2_R is strongly expressed throughout the human heart, including the ventricles, atrium, septum, apex, and Purkinje fiber cells [10, 30–34]. Moreover, CysLT_1_R is present on monocyte and macrophages [35] whereas CysLT_2_R on myocytes and endothelial cells (ECs) [10, 30, 35]. In smooth muscle cells (SMCs), conflicting expression has been reported among species. Indeed, CysLT_2_R but not CysLT_1_R has been detected in human coronary artery SMCs [30], while rat aortic SMCs express greater amounts of CysLT_1_R protein compared with CysLT_2_R and the intracellular calcium increase, induced by LTD4, was inhibited by both the CysLT_1_R antagonist montelukast and the dual CysLT_1_R/CysLT_2_R antagonist BAYu9773 [36].

The interaction of CysLTs with their receptors and the degree of their activation modulate several effects that could be relevant for the development of CVDs (Figure 1). Indeed, CysLTs exert negative inotropic action on the myocardium and decrease coronary blood flows with no effect on heart rate [37–41]; moreover, they may mediate contraction through the CysLT receptors on the endothelium or SMCs, as well as relaxation, which is endothelium dependent [42]. Furthermore, CysLTs can also stimulate proliferation of arterial SMCs and promote P-selectin surface expression, von Willebrand factor secretion, and platelet-activating factor synthesis in cultured ECs [43–45].

## 3. CysLT Modifiers: Change in Focus

The pathophysiological role of LTs in several inflammatory conditions and, particularly, in asthma is well documented, and several molecules, named *LT modifiers*, able to interfere with the LT biosynthetic cascade or with the LT receptors, have been approved for the treatment of asthma [46].

However, asthma may not be a classical comorbidity of cardiovascular disease; LTs have been implicated as potential mediators of cardiovascular risk in other inflammatory diseases.

In studies of patients with chronic obstructive pulmonary disease (COPD), characterized by high level of CysLTs [47], the prevalence of ischemic heart disease is almost twofold higher compared with the general population [48]. Based on this evidence, short-time treatment with the FLAP inhibitor BAYx1005 (DG031) has been evaluated both in patients with COPD and in patients with a history of myocardial infarction [49, 50]. However, although both treatment protocols resulted in only modest inhibition of LTB4 concentrations, the overall results suggested a tendency for decrease of inflammatory markers [49, 50]. Recently, Hoxha and colleagues try to delineate the potential role of montelukast, the most described leukotriene receptor antagonist, in the treatment of cardiovascular diseases. Results from animal model studies [51–54] and from recent clinical trials [55, 56] show that montelukast, beyond its traditional use, can serve to prevent cardiovascular disease in humans and inhibit the atherosclerosis development in in vivo animal models suggesting a potential cardiovascular protective role [57]. Despite some limitations, all these studies provide an initial suggestion of a potential beneficial effect of an anti-LT treatment in cardiovascular disease; thus, there is a need for conducting clinical trials to assess the future role of these mediators in the CVD treatment.

## 4. Atherosclerosis

Atherosclerosis is a chronic inflammatory fibroproliferative process associated with several pathophysiological reactions within the vascular wall [58–60], characterized by (1) subendothelial oxidation of low-density lipoproteins (LDL); (2) infiltration of monocytes and their conversion to macrophages and lipid-laden foam cells; (3) accumulation of mast cells and other inflammatory cells; and (4) proliferation of smooth muscle cells and secretion of fibrous elements contributing to the growth of occlusive plaques [60]. This pathological condition can lead to myocardial infarction, stroke, and peripheral occlusive vascular diseases [61].

**Figure 2:**
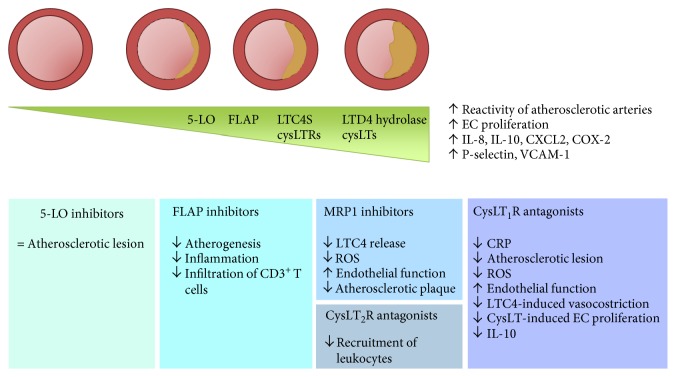
Involvement of the 5-LO pathway in the pathogenesis and progression of atherosclerosis and major effects of CysLT modifiers in humans and in experimental models.

In human atherosclerotic lesions, increased expression of the 5-LO pathway mediators and products, including 5-LO, FLAP, LTD4 hydrolase, LTC4S, LTB4, CysLTs, and CysLT receptors, was detectable [62, 63], suggesting the 5-LO pathway as a potential target for atheroprotective therapy (Figure 2).

The 5-LO-positive cells dramatically increased in advanced atherosclerotic lesions with progression from early to late stage of atherogenesis [63], and its expression has been mostly localized to macrophages which represent one major source of 5-LO [64], suggesting a possible role of 5-LO and its products in promoting lesion development [65].

In particular, a number of histochemical studies [63–66] pointed out that 5-LO was mostly present in activated CD68^+^ macrophages [63, 64] and that their distribution in lesions/plaque/aneurysmal arteries was not uniform. Indeed, the 5LO-positive cells were often observed at sites most prone to rupture [67], such as in the shoulder region below the fibrous cap, in the adventitia of diseased human arteries [63], in areas of neoangiogenesis, in granulomas around aneurysmal arteries [66], and also in neutrophilic granulocytes, dendritic, foam and mast cells [63].

From the time when the concept of inflammation and atherosclerosis was raised, a number of inflammatory mediators have been explored as potential therapeutic targets in this disease [68] and, among these, leukotrienes also have been investigated [35].

Although there is a long tradition of treating asthma with anti-CysLTs [69] and asthma may not be a classical comorbidity of atherosclerosis, some interesting indications were obtained from a randomized controlled trial of placebo versus the CysLT_1_R antagonist montelukast, which reported significantly lower levels of C-reactive protein in treated patients with severe asthma [55]. Although no follow-up of those patients was performed in terms of cardiovascular disease, the systemic anti-inflammatory effect of montelukast could provide an initial suggestion of a potential anti-CysLT beneficial effect in atherosclerosis [70]. In fact, periodontal disease that could be ascribed as one of the sources of chronic inflammation is associated with an increased risk of stroke [71], myocardial infarction [72], and the development of early atherosclerotic lesions in the carotid artery [73]. In a study, it was found that subjects with atherosclerotic plaques and increased carotid artery wall thickness had significantly elevated concentrations of CysLTs in their gingival crevicular fluid as compared with subjects without a visible plaque [74].

In addition to the studies implicating CysLTs in comorbidities of atherosclerosis, genetic and pharmacological experimental studies suggest the existence of a potential link between the CysLT signaling cascade and the pathogenesis/progression of atherosclerosis as well as its serious consequences such as myocardial infarction, brain ischemia, aortic aneurysms, and intimal hyperplasia [35, 75].

It was reported [76] that the identification of a locus on murine chromosome 6 that confers almost total resistance to atherogenesis and 5-LO was among the chromosome 6 locus candidates tested. The results showed that, in a congenic strain containing the resistant chromosome 6 (CON6), the mRNA levels of 5-LO and similarly 5-LO protein were reduced about 5-fold compared with the background strain.

A significant reduction in aortic lesions (more than 26-fold) observed in 5-LO^+/−^/LDLR^−/−^ mice compared to 5-LO^+/+^/LDLR^−/−^ mice further provides evidence of the involvement of 5-LO in the development of atherosclerotic lesions [65]. Moreover, it was reported that CON6 mice expressed a considerably reduced amount of 5-LO also in bone marrow and peritoneal monocytes/macrophages and that transplantation of CON6 or 5-LO^+/−^ bone marrow to LDLR^−/−^ mice had a similar effect on atherosclerosis (2- to 3-fold decrease) suggesting that the level of 5-LO in macrophages is responsible, at least in part, for the progression of atherosclerosis [65].

In addition, it was found that 5-LO genomic sequences of CON6 mice presented 2 nucleotide exchanges in the coding conserved region, which resulted into 2 amino acid exchanges of Ile-645 to Val (I645V) and of Val-646 to Ile (V646I) compared to wild-type mice, and that these murine mutations conferred an impaired 5-LO and LTA4S activity when introduced into the human enzyme [77].

A recent study [78] investigated the relationship between atorvastatin, a hydroxymethylglutaryl-CoA reductase inhibitor, and the 5-LO pathway mediators in an atherosclerotic rabbit model. New Zealand white rabbits subjected to carotid balloon dilation injury and treated with atorvastatin showed markedly lowered serum lipids and LTD4 levels compared with the control group. Similarly, mRNA expression of FLAP and CysLT_1_R was significantly inhibited by atorvastatin. Moreover, atorvastatin treatment stabilized carotid plaque and decreased vascular inflammation as demonstrated by a thickened elastic layer, less neointima hyperplasia, and macrophage proliferation. This study suggested that atorvastatin might stabilize carotid plaque by regulating the 5-LO pathway in atherosclerotic rabbits and delay the progression of atherosclerosis by exerting anti-inflammatory effects. In contrast, high-dose simvastatin treatment induced overexpression of FLAP in patients' muscle [79] and two explanations are possible for the conflicting results: they could be attributable to the dosage forms of statins or species differences.

In human, the Carotid Atherosclerosis Progression Study [80] examined whether polymorphisms in 8 genes related to the 5-LO pathway were associated with early atherosclerosis and remodeling as measured by IMT. The results showed that these genetic variants had little effect on early atherosclerosis and remodeling risk. However, the subjects enrolled in this study represent a community population with predominantly early atherosclerosis, and there were insufficient advanced plaque and stenosis to exclude associations with advanced atherosclerosis.

Previously, a randomly sampled cohort of healthy subjects identified two variants of 5-LO genotypes (lacking the common allele) that were accompanied by a significant increase in IMT and atherosclerotic plaques [81]. In this population, dietary arachidonic acid significantly enhanced the apparent atherogenic genotype effects and it was blunted by increased dietary intake of marine *n* − 3 fatty acids (which reduced the production of LTs) suggesting diet-gene interactions.

On the contrary, in apolipoprotein E-deficient (ApoE^−/−^) mice with either genetic (5-LO^−/−^) or pharmacological (L-739,010) inhibition of the 5-LO and subjected to atherosclerotic regimen with either an 8-week Paigen or 6-month Western diet, any difference in atherosclerotic lesion size was observed between the groups [82]. Moreover, the composition of advanced lesions did not indicate an effect on plaque stability as a result of 5-LO gene inactivation [82]. Another study on ApoE^−/−^/5-LO^−/−^ mice on a normal or Western diet showed no difference in atherosclerotic lesions compared to the control mice [66].

Despite 5-LO having to show a role in predisposition to atherosclerosis, taken together, all these controversial results do not clarify its role in the progression of pathology.

More convincing evidences of the involvement of the 5-LO pathway in atherosclerosis have been obtained evaluating FLAP [83]. In ApoE^−/−^/LDLR^−/−^ mice, the administration of two different FLAP inhibitors, MK-886 [84] and BAYx1005 [85], showed a reduction in atherogenesis. The beneficial effect of MK-866 was also confirmed in transgenic ApoE^−/−^ x CD4dnT*β*RII mice, with a dominant-negative TGF*β* type II receptor (dnTGF*β*RII) on CD4^+^ T cells, which displayed aggravated atherosclerosis. The treatment with MK-866 significantly reduced the aortic root lesion size and also inflammation, as CD3^+^ cells and IFN-*γ* mRNA levels [86].

This antiatherosclerotic effect was also reported for the CysLT_1_R antagonist. A reduction of atherosclerotic lesions in the aortic root was observed in ApoE^−/−^/LDLR^−/−^ mice treated with CysLT_1_R antagonist montelukast, even if in a lesser extent than FLAP inhibitors. This could be probably explained by “upstream” action of FLAP inhibitors on LT cascade, blocking both LTB4 and CysLT productions, while montelukast inhibits the cascade “downstream” by blocking only the effect of CysLTs and leaving LTB4 untouched [87]. However, as elucidated below, several studies established the effects of CysLT_1_R antagonists on atherosclerosis [54, 88–91].

The role of LTC4S has been investigated in the Muscatine study [92] which demonstrated the associations between coronary artery calcium (CAC) and intima/media thickness (IMT) (indices strongly associated with the amount of coronary atherosclerotic plaque) [93, 94] and the (−444) A > C promoter polymorphism of LTC4S in woman, but not in men.

A significant increase in LTC4S and CysLT_1_R gene expression was observed in a model of ApoE^−/−^ atherosclerotic heart disease subjected to hypoxic stress [54] compare to wild-type control mice. Moreover, LTC4S gene expression and activity and CysLT_1_R gene and protein expression were enhanced in ApoE^−/−^ mice after bouts of hypoxic stress. Administration of the CysLT_1_R antagonist montelukast reduced myocardial hypoxic areas suggesting a possible role of the CysLT pathway in oxygen supply. Accordingly, mRNA expression levels of LTC4S and CysLT_1_ were increased in human chronic ischemic compared to the nonischemic myocardium, suggesting similar mechanisms to those observed in mice [54].

The multidrug resistance protein-1 (MRP1) was suggested as a mediator of the effect of LTC4 on atherosclerosis. This protein acts as a transporter to the extracellular compartment [95] for LTC4 as well as glutathione, oxidized glutathione, and estrogen [96] and is abundantly expressed in vascular SMCs and in human [97] and in the murine myocardium as well [98]. Its relevance in human health and disease has been deeply investigated [99], and it continues to be of considerable preclinical and clinical interest.

An *in vitro* study showed a proatherogenic mechanism mediated by MRP1 and LTC4: pharmacological inhibition of MRP1 and CysLT_1_R by MK571 and montelukast, respectively, reduced angiotensin II-induced ROS release in vascular SMCs [88]. Moreover, the *in vivo* study on atherosclerosis-prone ApoE^−/−^ mice, fed a high-cholesterol diet and treated with MK571 or montelukast for 6 weeks, showed a significant improvement in endothelial function and reduction of atherosclerotic plaque generation. These data represent an indirect proof of the MRP1 and LTC4 roles in the atherosclerotic processes, indicating them as potentially promising targets for atheroprotective therapy [88].

Within atherosclerotic lesions, CysLTs, which are produced by coronary arteries [100], can locally mediate vascular reactivity exerting their effects by an autocrine and paracrine signaling [101]. Indeed, in addition to their well-known bronchoconstrictor effect, CysLTs, acting on SMCs, are also potent vasoconstrictors as observed in the human lungs [7, 102].

The hemodynamic effects induced by the CysLTs were evaluated in a small study on 6 patients without significant stenosis on a coronary angiogram but in which cardiovascular risk factors and coronary atherosclerosis cannot be completely excluded. The coronary vascular resistance, systemic mean arterial blood pressure, and heart rate were evaluated during and after the intracoronary LTD4 administration (3 nmol bolus): no changes in resistance were observed during administration, while an increase was observed at 10 and 15 min after administration. Moreover, systemic mean arterial blood pressure initially decreased while heart rate was increased, returning to baseline after 10 and 1 min postinjection, respectively, suggesting that small doses of CysLTs induce both an early and transient fall in mean arterial pressure and a late increase in small coronary arteriolar resistance [103].

The urine levels of CysLTs increased in patients during and after acute myocardial infarction, unstable angina attacks [104], and coronary artery diseases both before and after coronary artery bypass surgery [105]. CysLT receptor subtypes are expressed in diseased human arteries, and hyperreactivity of atherosclerotic coronary arteries in response to LTC4 was found to be associated with the expression of CysLT receptors [89].

*In vitro* studies on nonatherosclerotic human coronary arteries showed the lack of CysLT-induced coronary vasoconstriction [89, 105], although CysLT_2_R mRNA expression can be detected in coronary artery SMCs [30]. In contrast, LTD4 and LTC4 induced contraction in atherosclerotic coronary arteries which is inhibited by the CysLT_1_R antagonist ICI198615 [89, 106] suggesting increased sensitivity to CysLTs during atherogenesis, probably due to an increased in the number of the binding site for LTD4 and LTC4 in atherosclerotic vessels [89, 105].

Furthermore, threefold higher levels of CysLT_1_R transcripts compared with CysLT_2_R transcripts were observed in atherosclerotic lesions from human carotid arteries [90] and an increased CysLT_1_R expression in the aorta was observed in atherosclerotic ApoE^−/−^ mice, compared with nonatherosclerotic mice [66].

A more recent study [107] showed colocalization of the CysLT_1_R protein with markers for SMCs in human atherosclerotic lesions revealing also CysLT_1_R predominant perinuclear localization compared with cytoplasmatic alpha-smooth muscle actin localization. This study also showed an upregulation of CysLT_1_R induced by inflammatory conditions (LPS, L-6 and by prolonged exposure to IFN-*γ*). Taken together, all these observations suggest that a proinflammatory environment, such as atherosclerosis, may induce CysLT_1_R expression within the SMCs in the vascular wall and a major role of the CysLT_1_R in atherosclerosis compared to CysLT_2_R was observed.

Similar findings have been reported in EC, which under resting conditions exhibit a dominant CysLT_2_R, but in which a prolonged exposure to LPS or to proinflammatory cytokines upregulates CysLT_1_R expression [108]. Recently, an *in vitro* study [91] showed that LTC4 and LTD4 induce robust calcium influx in human umbilical vein endothelial cells (HUVECs), which was significantly inhibited by both Rho kinase inhibitor (Y27632) and CysLT_2_R antagonist (BayCysLT2), but not by CysLT_1_R antagonist (MK571), suggesting that contraction of EC, induced by LTD4, was mediated only by CysLT_2_R [91]. LTC4 and LTD4 also stimulated EC proliferation, which was completely blocked by a MEK inhibitor (PD98059) and inhibited by MK571, indicating the CysLT_1_R role in EC proliferation. In the same study, CysLTs significantly increased the TNF*α*-induced expression of the adhesion molecule VCAM-1 and attachment of leukocytes to ECs. Notably, the recruitment of leukocytes was significantly attenuated by BayCysLT2 but not by MK571 [91].

Furthermore, LTC4 and LTD4 increased the expression of the adhesion molecule P-selectin in human ECs [45, 109]. This increase was not inhibited by CysLT_1_R antagonists, suggesting a CysLT_2_R-induced effect. In HUVECs, CysLT_2_R activation may also induce other proinflammatory effects through increased transcriptional activity [110]. Indeed, the LTD4-induced upregulation of IL-8, CXCL-2, and COX-2 was not inhibited by CysLT_1_R antagonist but seems to be sensitive to synergistic effects between CysLT2 and protease-activated (PAR-1) receptors. Taken together, these results suggest that CysLTs increase, in a CysLTR-depending manner, EC proliferation and expression of inflammatory genes involved in the recruitment and adhesion of leukocytes, which play a critical role in the etiology of atherosclerosis.

In addition to ECs and SMCs, also, T lymphocytes are involved in atherosclerosis, and despite the fact that these cells might not express CysLT receptors, CysLTs could potentially modulate adaptive immunological reactions by inducing the activation of antigen-presenting cells.

In a murine model of asthma, myeloid dendritic cells were shown to express CysLT_1_R, and LTD4 stimulation increased the production of the immunomodulatory cytokine IL-10, which was inhibited by treatment with CysLT_1_R antagonists [111].

It has been shown that interleukin IL-10 overexpression can inhibit fatty-streak formation in C57BL/6J mice fed an atherogenic diet containing chocolate [112, 113]. Furthermore, in LDLR^−/−^ mice, the overexpression of IL-10 by T cells induced a significant decrease in lesion size and necrotic core, inhibiting advanced atherosclerotic lesions [114]. Moreover, the accumulation of cholesterol and phospholipid oxidation products in the aorta was decreased by 50% to 80%, unrelated to plasma lipid or IL-10 levels [114]. In line, IL-10 deficiency in ApoE^−/−^ (IL-10^−/−^/ApoE^−/−^) mice increased atherosclerotic lesion size compared with ApoE^−/−^ control mice [115]. These studies indicated as the production of IL-10 induced by LTD4 could have a protective role in the atherosclerotic process and suggested that CysLT signaling may represent one possible regulator of immunomodulatory functions in atherosclerosis.

## 5. Myocardial Infarction

The possible involvement of LTs in the development of myocardial infarct damage has been of considerable interest within recent years. The genetic variants within the 5-LO pathway are associated with an increased risk of stroke and myocardial infarction (MI) [75]; moreover, the production of CysLTs increases in ischemia-reperfusion injury in both patients and animal models.

Because of their rapid metabolism and excretion, LTs are difficult to be measured accurately in blood [116, 117], although elevated plasma concentrations of these mediators have been reported after acute MI [104]. They influence, directly or indirectly, coronary vascular resistance, infarct size, pulmonary vascular resistance, bronchial tone, and renal vascular resistance; moreover, they are key regulators of inflammation and thus potential targets to influence healing after MI [5].

Development of MI injury is characterized by three phases: ischemic, reperfusion, and inflammatory. This last phase is characterized by increased expression of cell adhesion molecules, as well as leukocyte infiltration in a manner similar to those observed during inflammatory reaction [118]. In this process, the inflammatory cells, invading myocardial tissue after infarction, or their metabolic products, play a crucial role in the development of the damage and may participate in reperfusion injury [119]. The importance of leukocyte in cardiovascular disease has recently been reviewed [120], and several reports indicate a correlation between myocardial infarct size and the magnitude of leukocyte infiltration [121, 122]. Among a number of inflammatory mediators regulating leukocytes, LTs should be included. Indeed, LTs are necessary for the function and migration of leukocytes [91, 109]; moreover, LTs could play a role in the development of MI since they influence fibroblasts [30], increase contractility and proliferation of smooth muscle cells [89, 106, 123], and are also important for vascular permeability [124].

In animal models, experimental myocardial infarction causes elevated LT production in the damaged tissue and evidence suggests that 5-LO products exert a detrimental role in tissue recovery. Several investigators have pharmacologically tested the effect of lipoxygenase inhibitors on ischemic injury [125]; however, since results are controversial, a number of transgenic mice were studied to overcome the limitation of unspecific responses by pharmacological agents.

Adamek and colleagues [126] determined the response to ischemia/reperfusion injury in mice with targeted disruption of 5-LO. The 5-LO-deficient mice exhibit an increased neutrophil infiltration and proinflammatory gene expression within the infarction area compared with wild-type mice. Nevertheless, authors report that, despite an important role of 5-LO in inflammatory responses, 5-LO seems to not play a major role in ischemia-reperfusion injury in the heart. These data compared with investigation made in other organs [127–129] hypothesize that 5-LO effects might be organ specific. However, although these results raise a doubt on to the role of LTs in myocardial ischemia, a number of evidence from other studies support their strong involvement.

Recently, in a large Danish cohort study [130, 131], the association between 20 preselected single-nucleotide polymorphisms (SNPs) and MI events has been evaluated, demonstrating that some common SNPs in the 5-lipoxygenase pathway were modestly associated with incident MI, suggesting a potential role for this pathway in the development of cardiovascular disease.

As reported for atherosclerosis, MRP1 seems to mediate, at least in part, the cardiac effects of LTC4. A recent study has suggested an important role of MRP1 on intracellular redox homeostasis and myocardial performance [51]. In this study, the cardiac effects of CysLT_1_R blocker montelukast and MRP1-inhibitor MK571 as well as MRP1 depletion were tested *in vitro* and *in vivo*. Results demonstrated that pharmacological blockade of CysLT_1_R prevents LTC4-induced ROS production and release in cultured cardiomyocytes and, additionally, that montelukast reduces oxidative stress and apoptosis in cardiomyocytes having a beneficial effect on myocardium remodeling and improves myocardial function after left ventricular injury in a mouse model of crio-induced MI. Moreover, the inhibition of LTC4 transport, either in MRP1^−/−^ mice or in MK571-mediated mice, resulted, *in vivo*, in reduced oxidative stress and apoptosis and demonstrated beneficial effects on cardiac remodeling after injury.

On the contrary, the role of LTD4 in MI is not clearly elucidated. LTD4 is one of the leukocyte metabolites with high coronary constrictor potency, mainly released from macrophages [132, 133] but also produced by a variety of tissues, including coronary and pulmonary arteries [134]. In a model of coronary stenosis and myocardial ischemia, LTD4 induced coronary constriction [135]; moreover, it was reported that its levels increased in infarcted rabbit hearts [136] and in urine of humans with acute cardiac ischemia [104]. LTD4 acted also as potent coronary vasoconstrictor in the isolated rat heart model, and this effect was more potent in chronically infarcted heart [137]. Intravenous administration of LTD4 produced prominent cardiovascular alteration in rat and dog, characterized by a decrease in blood pressure and a reduction in aortic arterial blood flow and stroke volume. Nevertheless, the administration in rat and dog of LY203647, described as a potent and selective antagonist of responses to both LTD4 and LTE4, did not alter the magnitude of myocardial ischemia [138]. The limitation of the influence of the endogenously produced LTD4 in the progression of cardiac damage was also confirmed in another study where an alternative specific antagonist L-660,711 had no effect on coronary blood flow and cardiac performance in rats following MI [137].

Aforementioned, the CysLTs exert their effects by binding to G-protein-coupled receptors CysLT_1_R and CysLT_2_R and novels GPR99 [12] and GPR17 [11].

To clarify which receptor was mediating the most of the cardiovascular CysLT effects, the consequences of the HAMI3379 and zafirlukast, CysLT_2_R and CysLT_1_R antagonists, respectively, were tested on LTC4-treated, Langendorff-perfused, guinea pig hearts [139]. Results showed that HAMI3379 was an effective antagonist of the cardiac effects of LTC4, while zafirlukast was found to be inactive in this experimental setting, suggesting that the cardiac CysLT effects are mainly mediated by CysLT_2_R and these results were in good agreement to the high expression of the CysLT_2_R in the heart and blood vessels. In another study [140], the treatment with BayCysLT_2_, a potent CysLT_2_R antagonist, attenuated increased infarction damage when administered either before ischemia or after reperfusion. This treatment prevented the increases in cell adhesion molecule gene expression and leukocyte infiltration into the myocardium, both hallmarks of the acute inflammatory response following MI. These findings indicate that CysLT_2_R activation results in heightened facilitation of diapedesis, which enhances the magnitude of the inflammatory response leading to additional damage to the site of injury [140]. This mechanism was then confirmed by an *in vivo* study [124].

Using the CysLT_2_R transgenic mice, overexpressing human CysLT_2_R in vascular endothelium, as well as knockout mice, a role for the CysLT_2_R in vascular permeability and myocardial ischemia/reperfusion injury has been shown [33, 34, 141]. In particular, the endothelial overexpression of CysLT_2_R [141] increased cardiomyocyte apoptosis in the peri-infarct region and induced an exacerbation of damage after MI resulting, from signaling through this receptor, in an increase in CD45^+^ cell infiltration, intermyofibrillal erythrocyte accumulation, and fluid extravasation worsening inflammatory gene expression and increasing infarct size.

On the contrary, the overexpression of CysLT_2_R also unaltered left ventricular function in uninjured myocardium [141].

The mechanism of action was partially explained in another study on the same model by demonstrating that CysLT_2_R mediates inflammatory reactions in a vascular bed-specific manner by altering transendothelial vesicle transport-based vascular permeability [34]. A very recent paper indicated the existence of endothelial and nonendothelial CysLT_2_R niches having separate roles in mediating inflammatory responses in which activation is required for injury exacerbation [124]. Particularly, endothelial receptor activation results in increased vascular permeability and leukocyte slow rolling, facilitating leukocyte transmigration, whereas nonendothelial receptors, likely located on resident/circulating leukocytes, facilitate leukocyte recruitment to the site of injury and activation of endothelial receptor.

GPR17 is a P2Y-like receptor responding to both uracil nucleotides and LTD4/LTC4 whose presence characterizes various organs susceptible to ischemic damage such as brain, kidney, and heart [11]. Moreover, it can interact with other closely related receptors, since its ability to act as a negative regulator of the CysLT_1_R [142, 143] was recently reported, as previously hypothesized by Maekawa and collaborators both *in vitro* and *in vivo* [8, 144, 145]. In normal mice, it was found expressed in cardiac-resident stromal cells [146] suggesting the same role observed in the central nervous system, where GPR17^+^ cells seem to have a role of a damage “sensor” able to activate healing program [147], whereas, following MI, GPR17 was found in resident and recruited CD45^+^ cells [146]. Interestingly, it was found that the treatment of the cardiac stromal cells with LTD4 exerted a potent chemotactic effect via GPR17 activation and that this effect can be reverted by cotreatment with montelukast, a GPR17 pharmacological antagonist [146]. These findings point to a specific GPR17 role in chemotactic guidance of stromal cells towards the ischemic sites and open to the hypothesis that the selective modulation of GPR17 signaling translates into beneficial treatments potentially reducing the extent of myocardial fibrosis and limiting the functional consequences of heart ischemia.

## 6. Summary

Cysteinyl leukotrienes are lipid mediators inducing proinflammatory signaling through the activation of specific receptors.

Exciting preclinical and clinical data indicate that the 5-LO pathway becomes activated in cardiovascular diseases and suggests an important role of CysLTs in atherosclerosis and in its ischemic complications such as myocardial infarction and stroke. Moreover, CysLT modifiers, generally safe and well tolerated, approved for the treatment of asthma, show significant cardioprotection in the experimental setting. To date, the information available give emphasis to CysLTs as potential targets in cardiovascular diseases and may provide the necessary background and justification to launch novel therapeutic programs. Nevertheless, further experimental and clinical studies are needed to determine the potential of therapeutic strategies targeting the 5-LO pathway in cardiovascular disease and the link existing between the human genetics and the 5-LO pathway in the inflammatory pathology of cardiovascular diseases.

## References

[1] Capra V., Thompson M. D., Sala A., Cole D. E., Folco G., Rovati G. E. (2007). Cysteinyl-leukotrienes and their receptors in asthma and other inflammatory diseases: critical update and emerging trends. *Medicinal Research Reviews*.

[2] Capra V. (2004). Molecular and functional aspects of human cysteinyl leukotriene receptors. *Pharmacological Research*.

[3] Funk C. D. (2001). Prostaglandins and leukotrienes: advances in eicosanoid biology. *Science*.

[4] Miller D. K., Gillard J. W., Vickers P. J. (1990). Identification and isolation of a membrane protein necessary for leukotriene production. *Nature*.

[5] Peters-Golden M., Henderson W. R. (2007). Leukotrienes. *The New England Journal of Medicine*.

[6] Brink C., Dahlen S. E., Drazen J. (2003). International Union of Pharmacology XXXVII. Nomenclature for leukotriene and lipoxin receptors. *Pharmacological Reviews*.

[7] Back M. (2002). Functional characteristics of cysteinyl-leukotriene receptor subtypes. *Life Sciences*.

[8] Maekawa A., Kanaoka Y., Xing W., Austen K. F. (2008). Functional recognition of a distinct receptor preferential for leukotriene E4 in mice lacking the cysteinyl leukotriene 1 and 2 receptors. *Proceedings of the National Academy of Sciences of the United States of America*.

[9] Lynch K. R., O'Neill G. P., Liu Q. (1999). Characterization of the human cysteinyl leukotriene CysLT1 receptor. *Nature*.

[10] Heise C. E., O'Dowd B. F., Figueroa D. J. (2000). Characterization of the human cysteinyl leukotriene 2 receptor. *The Journal of Biological Chemistry*.

[11] Ciana P., Fumagalli M., Trincavelli M. L. (2006). The orphan receptor GPR17 identified as a new dual uracil nucleotides/cysteinyl-leukotrienes receptor. *The EMBO Journal*.

[12] Kanaoka Y., Maekawa A., Austen K. F. (2013). Identification of GPR99 protein as a potential third cysteinyl leukotriene receptor with a preference for leukotriene E4 ligand. *The Journal of Biological Chemistry*.

[13] Paruchuri S., Jiang Y., Feng C., Francis S. A., Plutzky J., Boyce J. A. (2008). Leukotriene E4 activates peroxisome proliferator-activated receptor gamma and induces prostaglandin D2 generation by human mast cells. *The Journal of Biological Chemistry*.

[14] Paruchuri S., Tashimo H., Feng C. (2009). Leukotriene E4-induced pulmonary inflammation is mediated by the P2Y12 receptor. *The Journal of Experimental Medicine*.

[15] Lam B. K., Penrose J. F., Freeman G. J., Austen K. F. (1994). Expression cloning of a cDNA for human leukotriene C4 synthase, an integral membrane protein conjugating reduced glutathione to leukotriene A4. *Proceedings of the National Academy of Sciences of the United States of America*.

[16] Drazen J. M., Austen K. F. (1987). Leukotrienes and airway responses. *The American Review of Respiratory Disease*.

[17] Thien F. C., Walters E. H. (1995). Eicosanoids and asthma: an update. *Prostaglandins, Leukotrienes, and Essential Fatty Acids*.

[18] Feinmark S. J., Cannon P. J. (1986). Endothelial cell leukotriene C4 synthesis results from intercellular transfer of leukotriene A4 synthesized by polymorphonuclear leukocytes. *The Journal of Biological Chemistry*.

[19] Maclouf J. A., Murphy R. C. (1988). Transcellular metabolism of neutrophil-derived leukotriene A4 by human platelets. A potential cellular source of leukotriene C4. *The Journal of Biological Chemistry*.

[20] Dahinden C. A., Clancy R. M., Gross M., Chiller J. M., Hugli T. E. (1985). Leukotriene C4 production by murine mast cells: evidence of a role for extracellular leukotriene A4. *Proceedings of the National Academy of Sciences of the United States of America*.

[21] Bigby T. D., Meslier N. (1989). Transcellular lipoxygenase metabolism between monocytes and platelets. *Journal of Immunology*.

[22] Bigby T. D., Lee D. M., Meslier N., Gruenert D. C. (1989). Leukotriene A4 hydrolase activity of human airway epithelial cells. *Biochemical and Biophysical Research Communications*.

[23] Grimminger F., Sibelius U., Seeger W. (1991). Amplification of LTB4 generation in AM-PMN cocultures: transcellular 5-lipoxygenase metabolism. *The American Journal of Physiology*.

[24] Brady H. R., Serhan C. N. (1992). Adhesion promotes transcellular leukotriene biosynthesis during neutrophil-glomerular endothelial cell interactions: inhibition by antibodies against CD18 and L-selectin. *Biochemical and Biophysical Research Communications*.

[25] Iversen L., Kristensen P., Gron B., Ziboh V. A., Kragballe K. (1994). Human epidermis transforms exogenous leukotriene A4 into peptide leukotrienes: possible role in transcellular metabolism. *Archives of Dermatological Research*.

[26] Amat M., Diaz C., Vila L. (1998). Leukotriene A4 hydrolase and leukotriene C4 synthase activities in human chondrocytes: transcellular biosynthesis of leukotrienes during granulocyte-chondrocyte interaction. *Arthritis and Rheumatism*.

[27] Maclouf J., Sala A., Rossoni G., Berti F., Muller-Peddinghaus R., Folco G. (1996). Consequences of transcellular biosynthesis of leukotriene C4 on organ function. *Haemostasis*.

[28] Rovati G. E., Capra V. (2007). Cysteinyl-leukotriene receptors and cellular signals. *Scientific World Journal*.

[29] Sarau H. M., Ames R. S., Chambers J. (1999). Identification, molecular cloning, expression, and characterization of a cysteinyl leukotriene receptor. *Molecular Pharmacology*.

[30] Kamohara M., Takasaki J., Matsumoto M. (2001). Functional characterization of cysteinyl leukotriene CysLT_2_ receptor on human coronary artery smooth muscle cells. *Biochemical and Biophysical Research Communications*.

[31] Nothacker H. P., Wang Z., Zhu Y., Reinscheid R. K., Lin S. H., Civelli O. (2000). Molecular cloning and characterization of a second human cysteinyl leukotriene receptor: discovery of a subtype selective agonist. *Molecular Pharmacology*.

[32] Takasaki J., Kamohara M., Matsumoto M. (2000). The molecular characterization and tissue distribution of the human cysteinyl leukotriene CysLT_2_ receptor. *Biochemical and Biophysical Research Communications*.

[33] Hui Y., Cheng Y., Smalera I. (2004). Directed vascular expression of human cysteinyl leukotriene 2 receptor modulates endothelial permeability and systemic blood pressure. *Circulation*.

[34] Moos M. P., Mewburn J. D., Kan F. W. (2008). Cysteinyl leukotriene 2 receptor-mediated vascular permeability via transendothelial vesicle transport. *The FASEB Journal*.

[35] Back M., Hansson G. K. (2006). Leukotriene receptors in atherosclerosis. *Annals of Medicine*.

[36] Mazzetti L., Franchi-Micheli S., Nistri S. (2003). The ACh-induced contraction in rat aortas is mediated by the Cys Lt_1_ receptor via intracellular calcium mobilization in smooth muscle cells. *British Journal of Pharmacology*.

[37] Singh R. K., Gupta S., Dastidar S., Ray A. (2010). Cysteinyl leukotrienes and their receptors: molecular and functional characteristics. *Pharmacology*.

[38] Feuerstein G. (1984). Leukotrienes and the cardiovascular system. *Prostaglandins*.

[39] Lefer A. M. (1988). Thromboxane A2 and leukotrienes are eicosanoid mediators of shock and ischemic disorders. *Progress in Clinical and Biological Research*.

[40] Letts L. G. (1987). Leukotrienes: role in cardiovascular physiology. *Cardiovascular Clinics*.

[41] Folco G., Rossoni G., Buccellati C., Berti F., Maclouf J., Sala A. (2000). Leukotrienes in cardiovascular diseases. *American Journal of Respiratory and Critical Care Medicine*.

[42] Walch L., Norel X., Gascard J. P., Brink C. (2000). Functional studies of leukotriene receptors in vascular tissues. *American Journal of Respiratory and Critical Care Medicine*.

[43] McIntyre T. M., Zimmerman G. A., Prescott S. M. (1986). Leukotrienes C4 and D4 stimulate human endothelial cells to synthesize platelet-activating factor and bind neutrophils. *Proceedings of the National Academy of Sciences of the United States of America*.

[44] Datta Y. H., Romano M., Jacobson B. C., Golan D. E., Serhan C. N., Ewenstein B. M. (1995). Peptido-leukotrienes are potent agonists of von Willebrand factor secretion and P-selectin surface expression in human umbilical vein endothelial cells. *Circulation*.

[45] Pedersen K. E., Bochner B. S., Undem B. J. (1997). Cysteinyl leukotrienes induce P-selectin expression in human endothelial cells via a non-CysLT1 receptor-mediated mechanism. *The Journal of Pharmacology and Experimental Therapeutics*.

[46] Riccioni G., Bucciarelli T., Mancini B., Di Ilio C., D'Orazio N. (2007). Antileukotriene drugs: clinical application, effectiveness and safety. *Current Medicinal Chemistry*.

[47] de Prost N., El-Karak C., Avila M., Ichinose F., Vidal Melo M. F. (2011). Changes in cysteinyl leukotrienes during and after cardiac surgery with cardiopulmonary bypass in patients with and without chronic obstructive pulmonary disease. *The Journal of Thoracic and Cardiovascular Surgery*.

[48] Bäck M. (2008). Atherosclerosis, COPD and chronic inflammation. *Respiratory Medicine: COPD Update*.

[49] Gompertz S., Stockley R. A. (2002). A randomized, placebo-controlled trial of a leukotriene synthesis inhibitor in patients with COPD. *Chest*.

[50] Hakonarson H., Thorvaldsson S., Helgadottir A. (2005). Effects of a 5-lipoxygenase–activating protein inhibitor on biomarkers associated with risk of myocardial infarction: a randomized trial. *JAMA*.

[51] Becher U. M., Ghanem A., Tiyerili V., Furst D. O., Nickenig G., Mueller C. F. (2011). Inhibition of leukotriene C4 action reduces oxidative stress and apoptosis in cardiomyocytes and impedes remodeling after myocardial injury. *Journal of Molecular and Cellular Cardiology*.

[52] Daglar G., Karaca T., Yuksek Y. N. (2009). Effect of montelukast and MK-886 on hepatic ischemia-reperfusion injury in rats. *The Journal of Surgical Research*.

[53] Duran A., Otiuk H., Terzi E. H. (2013). Protective effect of montelukast, a cysteinyl leukotriene receptor-1 antagonist, against intestinal ischemia-reperfusion injury in the rat. *Acta Chirurgica Belgica*.

[54] Nobili E., Salvado M. D., Folkersen L. (2012). Cysteinyl leukotriene signaling aggravates myocardial hypoxia in experimental atherosclerotic heart disease. *PLoS One*.

[55] Allayee H., Hartiala J., Lee W. (2007). The effect of montelukast and low-dose theophylline on cardiovascular disease risk factors in asthmatics. *Chest*.

[56] Ingelsson E., Yin L., Back M. (2012). Nationwide cohort study of the leukotriene receptor antagonist montelukast and incident or recurrent cardiovascular disease. *The Journal of Allergy and Clinical Immunology*.

[57] Hoxha M., Rovati G. E., Cavanillas A. B. (2017). The leukotriene receptor antagonist montelukast and its possible role in the cardiovascular field. *European Journal of Clinical Pharmacology*.

[58] Witztum J. L. (1994). The oxidation hypothesis of atherosclerosis. *Lancet*.

[59] Ross R. (1999). Atherosclerosis—an inflammatory disease. *The New England Journal of Medicine*.

[60] Lusis A. J. (2000). Atherosclerosis. *Nature*.

[61] Rubin P., Mollison K. W. (2007). Pharmacotherapy of diseases mediated by 5-lipoxygenase pathway eicosanoids. *Prostaglandins & Other Lipid Mediators*.

[62] De Caterina R., Mazzone A., Giannessi D. (1988). Leukotriene B4 production in human atherosclerotic plaques. *Biomedica Biochimica Acta*.

[63] Spanbroek R., Grabner R., Lotzer K. (2003). Expanding expression of the 5-lipoxygenase pathway within the arterial wall during human atherogenesis. *Proceedings of the National Academy of Sciences of the United States of America*.

[64] Cipollone F., Mezzetti A., Fazia M. L. (2005). Association between 5-lipoxygenase expression and plaque instability in humans. *Arteriosclerosis, Thrombosis, and Vascular Biology*.

[65] Mehrabian M., Allayee H., Wong J. (2002). Identification of 5-lipoxygenase as a major gene contributing to atherosclerosis susceptibility in mice. *Circulation Research*.

[66] Zhao L., Moos M. P., Grabner R. (2004). The 5-lipoxygenase pathway promotes pathogenesis of hyperlipidemia-dependent aortic aneurysm. *Nature Medicine*.

[67] Falk E., Shah P. K., Fuster V. (1995). Coronary plaque disruption. *Circulation*.

[68] Hansson G. K. (2005). Inflammation, atherosclerosis, and coronary artery disease. *The New England Journal of Medicine*.

[69] Dahlen S. E. (2006). Treatment of asthma with antileukotrienes: first line or last resort therapy?. *European Journal of Pharmacology*.

[70] Back M. (2009). Leukotriene signaling in atherosclerosis and ischemia. *Cardiovascular Drugs and Therapy*.

[71] Grau A. J., Becher H., Ziegler C. M. (2004). Periodontal disease as a risk factor for ischemic stroke. *Stroke*.

[72] Rutger Persson G., Ohlsson O., Pettersson T., Renvert S. (2003). Chronic periodontitis, a significant relationship with acute myocardial infarction. *European Heart Journal*.

[73] Soder P. O., Soder B., Nowak J., Jogestrand T. (2005). Early carotid atherosclerosis in subjects with periodontal diseases. *Stroke*.

[74] Back M., Airila-Mansson S., Jogestrand T., Soder B., Soder P. O. (2007). Increased leukotriene concentrations in gingival crevicular fluid from subjects with periodontal disease and atherosclerosis. *Atherosclerosis*.

[75] Helgadottir A., Manolescu A., Thorleifsson G. (2004). The gene encoding 5-lipoxygenase activating protein confers risk of myocardial infarction and stroke. *Nature Genetics*.

[76] Mehrabian M., Wong J., Wang X. (2001). Genetic locus in mice that blocks development of atherosclerosis despite extreme hyperlipidemia. *Circulation Research*.

[77] Kuhn H., Anton M., Gerth C., Habenicht A. (2003). Amino acid differences in the deduced 5-lipoxygenase sequence of CAST atherosclerosis-resistance mice confer impaired activity when introduced into the human ortholog. *Arteriosclerosis, Thrombosis, and Vascular Biology*.

[78] Zhou G., Ge S., Liu D. (2010). Atorvastatin reduces plaque vulnerability in an atherosclerotic rabbit model by altering the 5-lipoxygenase pathway. *Cardiology*.

[79] Laaksonen R., Janis M. T., Oresic M. (2008). Lipidomics-based safety biomarkers for lipid-lowering treatments. *Angiology*.

[80] Bevan S., Lorenz M. W., Sitzer M., Markus H. S. (2009). Genetic variation in the leukotriene pathway and carotid intima-media thickness: a 2-stage replication study. *Stroke*.

[81] Dwyer J. H., Allayee H., Dwyer K. M. (2004). Arachidonate 5-lipoxygenase promoter genotype, dietary arachidonic acid, and atherosclerosis. *The New England Journal of Medicine*.

[82] Cao R. Y., St Amand T., Grabner R., Habenicht A. J., Funk C. D. (2009). Genetic and pharmacological inhibition of the 5-lipoxygenase/leukotriene pathway in atherosclerotic lesion development in ApoE deficient mice. *Atherosclerosis*.

[83] Poeckel D., Funk C. D. (2010). The 5-lipoxygenase/leukotriene pathway in preclinical models of cardiovascular disease. *Cardiovascular Research*.

[84] Jawien J., Gajda M., Rudling M. (2006). Inhibition of five lipoxygenase activating protein (FLAP) by MK-886 decreases atherosclerosis in apoE/LDLR-double knockout mice. *European Journal of Clinical Investigation*.

[85] Jawien J., Gajda M., Olszanecki R., Korbut R. (2007). BAY x 1005 attenuates atherosclerosis in apoE/LDLR - double knockout mice. *Journal of Physiology and Pharmacology*.

[86] Back M., Sultan A., Ovchinnikova O., Hansson G. K. (2007). 5-Lipoxygenase-activating protein: a potential link between innate and adaptive immunity in atherosclerosis and adipose tissue inflammation. *Circulation Research*.

[87] Jawien J., Gajda M., Wolkow P., Zuranska J., Olszanecki R., Korbut R. (2008). The effect of montelukast on atherogenesis in apoE/LDLR-double knockout mice. *Journal of Physiology and Pharmacology*.

[88] Mueller C. F., Wassmann K., Widder J. D. (2008). Multidrug resistance protein-1 affects oxidative stress, endothelial dysfunction, and atherogenesis via leukotriene C4 export. *Circulation*.

[89] Allen S., Dashwood M., Morrison K., Yacoub M. (1998). Differential leukotriene constrictor responses in human atherosclerotic coronary arteries. *Circulation*.

[90] Lotzer K., Spanbroek R., Hildner M. (2003). Differential leukotriene receptor expression and calcium responses in endothelial cells and macrophages indicate 5-lipoxygenase-dependent circuits of inflammation and atherogenesis. *Arteriosclerosis, Thrombosis, and Vascular Biology*.

[91] Duah E., Adapala R. K., Al-Azzam N. (2013). Cysteinyl leukotrienes regulate endothelial cell inflammatory and proliferative signals through CysLT_2_ and CysLT_1_ receptors. *Scientific Reports*.

[92] Iovannisci D. M., Lammer E. J., Steiner L. (2007). Association between a leukotriene C4 synthase gene promoter polymorphism and coronary artery calcium in young women: the Muscatine study. *Arteriosclerosis, Thrombosis, and Vascular Biology*.

[93] Allison M. A., Tiefenbrun J., Langer R. D., Wright C. M. (2005). Atherosclerotic calcification and intimal medial thickness of the carotid arteries. *International Journal of Cardiology*.

[94] Rumberger J. A., Simons D. B., Fitzpatrick L. A., Sheedy P. F., Schwartz R. S. (1995). Coronary artery calcium area by electron-beam computed tomography and coronary atherosclerotic plaque area. A histopathologic correlative study. *Circulation*.

[95] Kruh G. D., Zeng H., Rea P. A. (2001). MRP subfamily transporters and resistance to anticancer agents. *Journal of Bioenergetics and Biomembranes*.

[96] Mueller C. F., Widder J. D., McNally J. S., McCann L., Jones D. P., Harrison D. G. (2005). The role of the multidrug resistance protein-1 in modulation of endothelial cell oxidative stress. *Circulation Research*.

[97] Flens M. J., Zaman G. J., van der Valk P. (1996). Tissue distribution of the multidrug resistance protein. *The American Journal of Pathology*.

[98] Wijnholds J., Evers R., van Leusden M. R. (1997). Increased sensitivity to anticancer drugs and decreased inflammatory response in mice lacking the multidrug resistance-associated protein. *Nature Medicine*.

[99] Cole S. P. (2014). Multidrug resistance protein 1 (MRP1, ABCC1), a “multitasking” ATP-binding cassette (ABC) transporter. *The Journal of Biological Chemistry*.

[100] Piomelli D., Feinmark S. J., Cannon P. J. (1987). Leukotriene biosynthesis by canine and human coronary arteries. *The Journal of Pharmacology and Experimental Therapeutics*.

[101] Vannella K. M., McMillan T. R., Charbeneau R. P. (2007). Cysteinyl leukotrienes are autocrine and paracrine regulators of fibrocyte function. *Journal of Immunology*.

[102] Labat C., Ortiz J. L., Norel X. (1992). A second cysteinyl leukotriene receptor in human lung. *The Journal of Pharmacology and Experimental Therapeutics*.

[103] Vigorito C., Giordano A., Cirillo R., Genovese A., Rengo F., Marone G. (1997). Metabolic and hemodynamic effects of peptide leukotriene C4 and D4 in man. *International Journal of Clinical & Laboratory Research*.

[104] Carry M., Korley V., Willerson J. T., Weigelt L., Ford-Hutchinson A. W., Tagari P. (1992). Increased urinary leukotriene excretion in patients with cardiac ischemia. In vivo evidence for 5-lipoxygenase activation. *Circulation*.

[105] Allen S. P., Sampson A. P., Piper P. J., Chester A. H., Ohri S. K., Yacoub M. H. (1993). Enhanced excretion of urinary leukotriene E4 in coronary artery disease and after coronary artery bypass surgery. *Coronary Artery Disease*.

[106] Allen S. P., Dashwood M. R., Chester A. H. (1993). Influence of atherosclerosis on the vascular reactivity of isolated human epicardial coronary arteries to leukotriene C4. *Cardioscience*.

[107] Eaton A., Nagy E., Pacault M., Fauconnier J., Back M. (2012). Cysteinyl leukotriene signaling through perinuclear CysLT_1_ receptors on vascular smooth muscle cells transduces nuclear calcium signaling and alterations of gene expression. *Journal of Molecular Medicine (Berlin, Germany)*.

[108] Gronert K., Martinsson-Niskanen T., Ravasi S., Chiang N., Serhan C. N. (2001). Selectivity of recombinant human leukotriene D_4_, leukotriene B_4_, and lipoxin A_4_ receptors with aspirin-triggered 15-epi-LXA_4_ and regulation of vascular and inflammatory responses. *The American Journal of Pathology*.

[109] Papayianni A., Serhan C. N., Brady H. R. (1996). Lipoxin A4 and B4 inhibit leukotriene-stimulated interactions of human neutrophils and endothelial cells. *Journal of Immunology*.

[110] Uzonyi B., Lotzer K., Jahn S. (2006). Cysteinyl leukotriene 2 receptor and protease-activated receptor 1 activate strongly correlated early genes in human endothelial cells. *Proceedings of the National Academy of Sciences*.

[111] Machida I., Matsuse H., Kondo Y. (2004). Cysteinyl leukotrienes regulate dendritic cell functions in a murine model of asthma. *Journal of Immunology*.

[112] Mallat Z., Besnard S., Duriez M. (1999). Protective role of interleukin-10 in atherosclerosis. *Circulation Research*.

[113] Pinderski Oslund L. J., Hedrick C. C., Olvera T. (1999). Interleukin-10 blocks atherosclerotic events in vitro and in vivo. *Arteriosclerosis, Thrombosis, and Vascular Biology*.

[114] Pinderski L. J., Fischbein M. P., Subbanagounder G. (2002). Overexpression of interleukin-10 by activated T lymphocytes inhibits atherosclerosis in LDL receptor-deficient mice by altering lymphocyte and macrophage phenotypes. *Circulation Research*.

[115] Caligiuri G., Rudling M., Ollivier V. (2003). Interleukin-10 deficiency increases atherosclerosis, thrombosis, and low-density lipoproteins in apolipoprotein E knockout mice. *Molecular Medicine*.

[116] Koller M., Schonfeld W., Knoller J. (1985). The metabolism of leukotrienes in blood plasma studied by high-performance liquid chromatography. *Biochimica et Biophysica Acta*.

[117] Okubo T., Takahashi H., Sumitomo M., Shindoh K., Suzuki S. (1987). Plasma levels of leukotrienes C4 and D4 during wheezing attack in asthmatic patients. *International Archives of Allergy and Applied Immunology*.

[118] Fishbein M. C., Maclean D., Maroko P. R. (1978). Experimental myocardial infarction in the rat: qualitative and quantitative changes during pathologic evolution. *The American Journal of Pathology*.

[119] Romson J. L., Hook B. G., Kunkel S. L., Abrams G. D., Schork M. A., Lucchesi B. R. (1983). Reduction of the extent of ischemic myocardial injury by neutrophil depletion in the dog. *Circulation*.

[120] Swirski F. K., Nahrendorf M. (2013). Leukocyte behavior in atherosclerosis, myocardial infarction, and heart failure. *Science*.

[121] Engler R. L., Schmid-Schonbein G. W., Pavelec R. S. (1983). Leukocyte capillary plugging in myocardial ischemia and reperfusion in the dog. *The American Journal of Pathology*.

[122] Jolly S. R., Kane W. J., Hook B. G., Abrams G. D., Kunkel S. L., Lucchesi B. R. (1986). Reduction of myocardial infarct size by neutrophil depletion: effect of duration of occlusion. *American Heart Journal*.

[123] Porreca E., Di Febbo C., Di Sciullo A. (1996). Cysteinyl leukotriene D4 induced vascular smooth muscle cell proliferation: a possible role in myointimal hyperplasia. *Thrombosis and Haemostasis*.

[124] Ni N. C., Ballantyne L. L., Mewburn J. D., Funk C. D. (2014). Multiple-site activation of the cysteinyl leukotriene receptor 2 is required for exacerbation of ischemia/reperfusion injury. *Arteriosclerosis, Thrombosis, and Vascular Biology*.

[125] Gross G. J., Falck J. R., Gross E. R., Isbell M., Moore J., Nithipatikom K. (2005). Cytochrome P450 and arachidonic acid metabolites: role in myocardial ischemia/reperfusion injury revisited. *Cardiovascular Research*.

[126] Adamek A., Jung S., Dienesch C. (2007). Role of 5-lipoxygenase in myocardial ischemia-reperfusion injury in mice. *European Journal of Pharmacology*.

[127] Kitagawa K., Matsumoto M., Hori M. (2004). Cerebral schemia in 5-lipoxygenase knockout mice. *Brain Research*.

[128] Chatterjee P. K., Patel N. S., Cuzzocrea S. (2004). The cyclopentenone prostaglandin 15-deoxy-∆^12,14^-prostaglandin J2 ameliorates ischemic acute renal failure. *Cardiovascular Research*.

[129] Cuzzocrea S., Rossi A., Serraino I. (2003). 5-Lipoxygenase knockout mice exhibit a resistance to splanchnic artery occlusion shock. *Shock*.

[130] Gammelmark A., Lundbye-Christensen S., Tjonneland A., Schmidt E. B., Overvad K., Nielsen M. S. (2017). Interactions between 5-lipoxygenase polymorphisms and adipose tissue contents of arachidonic and eicosapentaenoic acids do not affect risk of myocardial infarction in middle-aged men and women in a Danish case-cohort study. *The Journal of Nutrition*.

[131] Gammelmark A., Nielsen M. S., Lundbye-Christensen S., Tjonneland A., Schmidt E. B., Overvad K. (2016). Common polymorphisms in the 5-lipoxygenase pathway and risk of incident myocardial infarction: a Danish case-cohort study. *PLoS One*.

[132] Burke J. A., Levi R., Guo Z. G., Corey E. J. (1982). Leukotrienes C4, D4 and E4: effects on human and guinea-pig cardiac preparations in vitro. *The Journal of Pharmacology and Experimental Therapeutics*.

[133] Scott W. A., Pawlowski N. A., Andreach M., Cohn Z. A. (1982). Resting macrophages produce distinct metabolites from exogenous arachidonic acid. *The Journal of Experimental Medicine*.

[134] Piper P. J., Letts L. G., Galton S. A. (1983). Generation of a leukotriene-like substance from porcine vascular and other tissues. *Prostaglandins*.

[135] Ertl G., Fiedler V. B., Bauer B., Schwarzenberger P., Kochsiek K. (1986). Effects of nifedipine and indomethacin on leukotriene C4- and D4-induced coronary constriction at normal and reduced coronary perfusion in dogs. *Journal of Cardiovascular Pharmacology*.

[136] Evers A. S., Murphree S., Saffitz J. E., Jakschik B. A., Needleman P. (1985). Effects of endogenously produced leukotrienes, thromboxane, and prostaglandins on coronary vascular resistance in rabbit myocardial infarction. *The Journal of Clinical Investigation*.

[137] Han H., Tian R., Neubauer S., Gaudron P., Hu K., Ertl G. (1994). Effects of LTD4 and its specific antagonist L-660,711 in isolated rat hearts with chronic myocardial infarction. *The American Journal of Physiology*.

[138] Hahn R. A., MacDonald B. R., Morgan E. (1992). Evaluation of LY203647 on cardiovascular leukotriene D4 receptors and myocardial reperfusion injury. *The Journal of Pharmacology and Experimental Therapeutics*.

[139] Wunder F., Tinel H., Kast R. (2010). Pharmacological characterization of the first potent and selective antagonist at the cysteinyl leukotriene 2 (CysLT_2_) receptor. *British Journal of Pharmacology*.

[140] Ni N. C., Yan D., Ballantyne L. L. (2011). A selective cysteinyl leukotriene receptor 2 antagonist blocks myocardial ischemia/reperfusion injury and vascular permeability in mice. *The Journal of Pharmacology and Experimental Therapeutics*.

[141] Jiang W., Hall S. R., Moos M. P. (2008). Endothelial cysteinyl leukotriene 2 receptor expression mediates myocardial ischemia-reperfusion injury. *The American Journal of Pathology*.

[142] Benned-Jensen T., Rosenkilde M. M. (2010). Distinct expression and ligand-binding profiles of two constitutively active GPR17 splice variants. *British Journal of Pharmacology*.

[143] Qi A. D., Harden T. K., Nicholas R. A. (2013). Is GPR17 a P2Y/leukotriene receptor? Examination of uracil nucleotides, nucleotide sugars, and cysteinyl leukotrienes as agonists of GPR17. *The Journal of Pharmacology and Experimental Therapeutics*.

[144] Maekawa A., Xing W., Austen K. F., Kanaoka Y. (2010). GPR17 regulates immune pulmonary inflammation induced by house dust mites. *Journal of Immunology*.

[145] Maekawa A., Balestrieri B., Austen K. F., Kanaoka Y. (2009). GPR17 is a negative regulator of the cysteinyl leukotriene 1 receptor response to leukotriene D4. *Proceedings of the National Academy of Sciences of the United States of America*.

[146] Cosentino S., Castiglioni L., Colazzo F. (2014). Expression of dual nucleotides/cysteinyl-leukotrienes receptor GPR17 in early trafficking of cardiac stromal cells after myocardial infarction. *Journal of Cellular and Molecular Medicine*.

[147] Ceruti S., Villa G., Genovese T. (2009). The P2Y-like receptor GPR17 as a sensor of damage and a new potential target in spinal cord injury. *Brain*.

